# Student Pharmacists’ Perceptions of Amazon Pharmacy

**DOI:** 10.3390/pharmacy9040166

**Published:** 2021-10-11

**Authors:** Alexandra Stich, Christian Cava, Dominic Cava, David R. Axon

**Affiliations:** Department of Pharmacy Practice and Science, College of Pharmacy, University of Arizona, Tucson, AZ 85721, USA; amstich@pharmacy.arizona.edu (A.S.); cacava@pharmacy.arizona.edu (C.C.); drcava@pharmacy.arizona.edu (D.C.)

**Keywords:** online pharmacy, qualitative research, semi-structured interviews, outcomes, pharmacy experience, pharmacy job market, student pharmacists

## Abstract

Amazon recently launched their online pharmacy in the United States (US). However, no studies have explored student pharmacists’ perceptions of the potential impact of Amazon Pharmacy. This qualitative study used individual semi-structured interviews to examine third- and fourth-year student pharmacists’ perceptions of how Amazon Pharmacy will affect economic, clinical, and humanistic outcomes; the pharmacy experience; and the job market. Interviews were audio-recorded, transcribed verbatim, and thematically analyzed by two independent reviewers until saturation was reached, with differences resolved through discussion with a third researcher. Seventeen students participated in the study. Five themes were identified: perceived economic outcomes for patients, perceived clinical outcomes for patients, perceived humanistic outcomes for patients, perceived impact of the pharmacy experience for patients, and perceived influence of Amazon Pharmacy on the pharmacy market. The majority suggested Amazon Pharmacy would offer lower costs for patients (71%), improved medication adherence (76%), and improved quality of life (65%). There was a consensus that the Amazon Pharmacy experience would be different, with various opinions highlighting potential positive or negative aspects of the service. There were mixed opinions about job opportunities and impact on existing pharmacies. Future studies should evaluate economic, clinical, and humanistic outcomes for patients utilizing Amazon Pharmacy.

## 1. Introduction

Amazon is a Fortune 500 company that has recently entered the online pharmaceutical market [1,2] following their recent acquisition of PillPack Inc. [3]. Amazon Pharmacy customers can access prescription information via the website, phone application, or telephone call [2]. Amazon Prime members not using prescription insurance are eligible to receive discounts on medications [2]. Additionally, customers can access pharmacists 24/7 online or via telephone, can opt for unit dose packaging, and can receive free two-day delivery [2,3].

The patient population that Amazon Pharmacy will serve is currently unknown. Previous studies have demonstrated that increased age, comorbidities, education beyond high school, and residing further from a brick-and-mortar pharmacy have been associated with utilizing mail-order services [4]. Online pharmacy services have been more frequently exploited by patients who have undergone polypharmacy, had increased medication costs, had middle-to-high economic status, and been prescribed erectile dysfunction medication [5]. In addition, African-American patients, Medicaid-insured patients, and patients prescribed narcotic medications have lower odds of online pharmacy use [5].

Patients desire safe and reliable prescription services [6]. Community pharmacists at brick-and-mortar stores are considered one of the most accessible healthcare resources for patient recommendations, counseling, and questions [6]. In addition, many patients value their relationship with their local community pharmacists [6]. However, Amazon Pharmacy, as an online platform, may be an appealing and convenient option for many patients, ranging from young millennials who may not have loyalty to specific pharmacies to elderly patients who have difficulty travelling to pharmacies. This is particularly pertinent in the current era of a global viral pandemic, where people are concerned about contracting the virus.

The perceived impact of Amazon Pharmacy on patient clinical outcomes is also unknown. Previous studies have found that patients who utilized mail-order pharmacies were more likely to achieve their target low-density lipoprotein cholesterol levels compared to those who use local pharmacies [7]. Patients with type II diabetes mellitus were found to have achieved a significantly higher proportion of days covered with mail-order services compared to patients who utilized a community pharmacy [8]. Additionally, emergency department visits and inpatient hospitalizations were found to be significantly reduced for patients in a mail-order group compared to those in a non-mail-order group, which the authors suggested was due to improved adherence in the mail-order pharmacy group [8].

Furthermore, the perceived impact of Amazon Pharmacy on the pharmacy job market is unknown. Amazon has the logistical capacity to provide pharmacy services across the country, popular name recognition, and substantial buying power to enhance price competitiveness [1] that other independent, mail-order, or retail pharmacies may not be able to accomplish.

There is therefore a need to investigate the perceived impact of Amazon Pharmacy on patients, the pharmacy experience, and the job market. There are no current studies regarding student pharmacists’ perceptions of the impact of Amazon in the pharmacy world. Since these students will be the future pharmacists adapting to Amazon’s influence, it is important to explore their perceptions of possible concerns, opinions, expectations, and opportunities associated with Amazon Pharmacy. This research team sought to conduct individual interviews with thematic analysis to create in-depth, thought-provoking conversations to highlight specific sets of themes common among students, as successfully achieved in previous studies [9,10,11,12].

The objective of this study was to examine third- and fourth-year student pharmacists’ perceptions of how Amazon Pharmacy will affect economic, clinical, and humanistic outcomes; the pharmacy experience; and the job market.

## 2. Materials and Methods

This was a qualitative study that utilized individual semi-structured online interviews with thematic analysis to elicit student pharmacists’ perceptions of Amazon Pharmacy. This approach was chosen to provide an opportunity for students to elaborate on their perceptions of Amazon Pharmacy while remaining focused on the topics of interest. This report followed the Consolidated Criteria for Reporting Qualitative Research (COREQ) [13].

A semi-structured interview guide was specifically developed for this study. It contained standardized instructions for interviewers and three core questions: (1) perceptions of Amazon Pharmacy regarding economic, clinical, and humanistic outcomes; (2) perceptions of Amazon Pharmacy on the pharmacy experience; and (3) perceptions of Amazon Pharmacy on the job market. Each core question was followed by appropriate probing questions that were asked as necessary (Table 1).

After the interview, participants completed an online demographic questionnaire that was created using Research Electronic Data Capture version 11.0.0 (Vanderbilt University, Nashville, TN, USA). The questionnaire contained four items: gender, previous pharmacy work experience (community, hospital, online, medication management center, or other), year in pharmacy program (third year or fourth year), and previous exposure/experience with Amazon Pharmacy (yes or no).

To be eligible for this study, participants had to be third- or fourth-year student pharmacist enrolled in the Doctor of Pharmacy (PharmD) program at the University of Arizona College of Pharmacy in the United States (n = 280 eligible students). An email was sent to all eligible students that invited them to participate in the study. Participants were offered a $10 gift card for their participation. All eligible individuals who indicated their interest in participating in the study were interviewed. For those who chose to participate, an interview was scheduled at a mutually convenient time.

The research team consisted of four trained researchers (D.R.A., A.S., C.C. and D.C.). The principal investigator (D.R.A. (male)) is an academic pharmacist with a PhD in administrative pharmacy and extensive experience in qualitative research methods. The remaining three researchers (A.S. (female), C.C. (male), and D.C. (male)) were student pharmacists who received appropriate training in qualitative methodologies from the principal investigator and worked under his supervision.

Before the interview began, the participant was introduced to the research team and given an overview of the study, reminded that their participation was voluntary, and asked to provide verbal informed consent. Participants were therefore made aware of the study purpose and had an existing relationship with the investigators because three were student pharmacist at the college where students were sampled and one was an academician who provided instruction to the students as part of the pharmacy curriculum. However, the academician who provided instruction to the students was not directly involved in recruiting participants or conducting the interviews so that the students did not feel compelled to participate for fear of this impacting their course scores. Individual audio-recorded interviews were conducted online via Zoom (Zoom Video Communications, San Jose, CA, USA) between April 2021 and May 2021. Interviews lasted for up to 30 min. During the interview, one investigator (A.S., C.C., or D.C.) interviewed the participant following the standardized semi-structured interview guide, asking each core question in turn with further probing questions as necessary. At the end of the interview, the participant was asked if they had any additional comments about Amazon Pharmacy to share. Another researcher (A.S., C.C., or D.C.) took notes during each interview to highlight key points. Only the researchers and the participant were present during the interview. No repeat interviews were conducted with the same participants.

Data analysis followed the framework method and involved the following steps: transcribing the interview, familiarizing oneself with the interview transcript, coding the transcript, developing and applying an analytical framework, charting the data into the framework, and finally interpreting the data [14]. One member of the research team (A.S.) transcribed each audio-recorded interview verbatim with the omission of any participant identifiers to maintain confidentiality. Transcripts were not returned to participants for comments. Two independent reviewers (C.C. and D.C.) performed qualitative thematic analysis using ATLAS.ti Windows software version 9 (ATLAS.ti Scientific Software Development GmbH, Berlin, Germany). The reviewers first performed open coding by reading the transcripts in their entirety to become familiar with their contents and to compare notes taken during the interview with the transcript. The reviewers made notes in the margin to highlight themes that occurred. The two reviewers then performed selective coding, in which they coded the data and grouped similar codes into themes. Then, they performed axial coding, in which they compared their lists for areas of similarities and differences. Differences were resolved through consensus with a third independent reviewer (A.S.), and inter-coder reliability was calculated. Finally, the reviewers performed selective coding to identify representative quotations for each theme. Analysis continued in an iterative manner until a saturation of ideas was met.

## 3. Results

### 3.1. Participants

A total of 17 student pharmacists participated in individual semi-structured interviews. Most participants were women (59%), were third-year students (53%), and had previous work experience in community pharmacy (88%). Approximately one-third had exposure/experience with Amazon Pharmacy (29%) (Table 2).

### 3.2. Thematic Analysis

Five themes related to Amazon Pharmacy were identified: (1) perceived economic outcomes for patients, (2) perceived clinical outcomes for patients, (3) perceived humanistic outcomes for patients, (4) perceived impact of pharmacy experience for patients, and (5) perceived influence of Amazon Pharmacy on the pharmacy market. The inter-coder reliability was 97%.

### 3.3. Theme 1: Perceived Economic Outcomes for Patients

Most student pharmacists indicated that Amazon Pharmacy would offer lower costs for patients (n = 12; 71%). Many student pharmacists suggested that Amazon could offer lower costs due to being a large, competitive company. One individual reasoned that people can purchase household items on Amazon for a cheaper cost, so Amazon medications should be cheaper as well.

*“I feel like there would be a benefit because Amazon is such a huge company, and they probably would have a lot of buying power as they get more into the field of pharmacy and negotiate lower prices for their customers.”*

(Participant 10)

Alternatively, four student pharmacists (24%) stated that Amazon Pharmacy would have similar medication costs to other pharmacies due to set copayments or limitations from patient insurance plans.

*“As long as it’s within their insurance plan, I don’t really see a difference from them getting it at CVS, Walgreens, or Amazon if it’s all going to end up being the same copay for them.”*

(Participant 6)

### 3.4. Theme 2: Perceived Clinical Outcomes for Patients

Most student pharmacists predicted an improvement in medication adherence (n = 13; 76%). Many explained that adherence improvements would apply for patients with chronic diseases that required lifetime medications. For instance, they mentioned that offering PillPack daily dose packaging and delivery to patient homes should ideally increase adherence. They expressed that providing patients with access to their medications in a timely manner through scheduled deliveries allows patients to readily have their medicine and be less likely to have gaps in therapy.

*“I think PillPack in itself increases adherence, and they deliver, so that’s going to make sure patients have their medications on them more easily than having to go to the pharmacy, so that in itself is going to increase clinical outcomes for patients.”*

(Participant 3)

Four participants (24%) perceived a reduction in adherence. They expressed perceived delays in receiving shipments and a lack of physical reminders to refill medications and take their medicine properly in comparison to patients who use brick and mortar pharmacies.

*“I know there’s also issues with mail orders and sometimes they won’t get their meds on time.”*

(Participant 6)

Nine student pharmacists perceived improved health control for patients (n = 9; 53%). Several participants acknowledged that one-on-one conversations with pharmacists on the phone could improve health control for patients by providing invaluable education and offering devoted time to problem-solve any barriers to medication adherence. Others mentioned that better adherence would lead to improved clinical outcomes for patients.

*“With Amazon they are forced to call and talk to the patient one on one, so it can improve clinical outcomes that way and it could be a net positive overall.”*

(Participant 5)

Those that foresaw a reduction in clinical outcomes pointed out that a loss of face-to-face interaction with pharmacists could lead to patients incorrectly using their medications. Specifically, they commented on a lack of hands-on training for medications or supplies that may be less user-friendly, such as diabetic equipment.

*“It would be less because you don’t have that in person experience with patients asking questions and if they need hands on experience with diabetes, like finger sticks or autoinjectors.”*

(Participant 5)

### 3.5. Theme 3: Perceived Humanistic Outcomes for Patients

Participants indicated that there could be an improvement or a reduction in patients’ quality of life. More individuals suggested an improvement (n = 11; 65%). The reasons for such an improvement included an ease of access to medications and not having to wait in line in a stressful and busy brick-and-mortar pharmacy.

*“Patients who do not have access to transportation or who are older and cannot drive, that’s really going to improve their quality of life.”*

(Participant 12)

Conversely, participants reported a possible reduction in patients’ quality of life due to missing out on a personable face-to-face relationship with community pharmacists. They mentioned that when patients value their relationships with their local pharmacists and enjoy their conversations, they are more likely to ask questions and get medical advice.

*“For patient’s quality of life, I think they’re losing a big resource if they switch to Amazon losing that face-to-face contact where you’re able to go in and check with your pharmacist and have that person that’s really accessible in the community setting.”*

(Participant 7)

### 3.6. Theme 4: Perceived Impact of Pharmacy Experience for Patients

Participants all agreed that the Amazon Pharmacy experience would be different (either better or worse) compared to other pharmacies. Several participants highlighted convenience and delivery as the forefront of the pharmacy experience for patients who use Amazon Pharmacy. They emphasized that the delivery system through the online platform is timely and consistent.

*“I think there’s a lot of pros to it as a patient ...getting your medications delivered right to your door so you’re not waiting...it’s going to be really nice.”*

(Participant 10)

Additionally, some student pharmacists portrayed an improvement in patient–pharmacist relationships due to extended hours for Amazon Pharmacy telephone services and the ease of asking pharmacists questions over the phone.

*“24-h line where you can call and ask pharmacists questions ...whereas typical pharmacies are open only certain hours.”*

(Participant 14)

Conversely, those who highlighted a worse outcome emphasized a gap in patient–pharmacist relationships (n = 13; 76%). Students perceived there would be less opportunities for in-person demonstrations for acute issues, less personalized service with more distant relationships with pharmacy staff, and a loss of routine interactions with health professionals.

*“It’s not like you can walk in and be like ‘hey I have this red eye thing can you look at this’? acute issues might not be able to be addressed.”*

(Participant 4)

Participants mentioned that patients may feel less comfortable talking with a stranger over the phone, have a reduced desire to ask questions, and may have to wait on hold for long time periods. One participant mentioned that patients may not speak with a pharmacist if technicians are the ones dedicated to answering the phones.

*“I think the pharmacist–patient relationship won’t be as personalized.”*

(Participant 5)

### 3.7. Theme 5: Perceived Influence of Amazon Pharmacy on the Pharmacy Market

Several participants indicated that Amazon Pharmacy could provide more job opportunities for future pharmacists within the company (n = 11; 65%). Students described that as a new business, there would be new job openings with additional opportunities as it expands.

*“I do think it’ll open up a lot more job opportunities for future pharmacists.”*

(Participant 11)

Other student pharmacists mentioned that Amazon Pharmacy could expand job availability within Amazon and at other pharmacies.

*“I think Amazon Pharmacy will increase job opportunities within the company itself and within other companies as other pharmacies look at Amazon’s model and start to incorporate it into their own.”*

(Participant 2)

Some participants mentioned that Amazon Pharmacy could offer more unconventional opportunities for new graduates, including job specialization.

*“They do provide a lot of different opportunities for pharmacists whether they want to be more on a community dispensing side and verifying, or on the other side, they have a lot of different departments to be involved with...like quality improvement and HR...a lot of different opportunities.”*

(Participant 14)

However, a few students mentioned that they expect the jobs may offer a lower pay. In addition, some student pharmacists indicated a potential reduction in job opportunities for future pharmacists if Amazon Pharmacy developed into a mass production center with a higher technician-to-pharmacist ratio. They perceived Amazon to have a “big business” mindset that could reduce costs and maximize efficiency through automation.

*“In the long run it’ll be more negative once people start working for this big business to optimize prescription ratios and increase the number of technicians to pharmacists.”*

(Participant 7)

Most participants indicated that Amazon Pharmacy may have an overall negative impact on other pharmacies, reasoning that Amazon is an innovative company and can offer a competitive price and service compared to other pharmacies. Many participants believed that job opportunities would decrease at other pharmacies if they lose patients to Amazon Pharmacy and consequently reduce staff costs.

*“I think Amazon Pharmacy would increase competitiveness with those other pharmacies and put selective pressure on...several businesses to close down if they can’t keep up with Amazon.”*

(Participant 9)

A smaller group of students indicated they did not know how Amazon Pharmacy would affect existing pharmacies or that there would not be a negative impact on existing pharmacies. Some students said that where patients can receive pharmacy services may be limited based on where their insurance plan is accepted. Others stated that there would not be a large impact on brick-and-mortar pharmacies due to the ability to adapt and the constant need for medications for acute conditions.

*“I think it will make other pharmacies expand their model to be competitive with Amazon so other pharmacies will start to adapt.”*

(Participant 3)

Additionally, independent pharmacies and other mail-order pharmacies may have services for a niche market whose patients may not seek Amazon Pharmacy services.

*“Independent pharmacies will find more unique niches for them to fill, so something to keep them competitive like compounding and specialty.”*

(Participant 1)

## 4. Discussion

The findings from these semi-structured interviews offer insight into the previously uninvestigated perceptions of third- and fourth-year student pharmacists at one college of pharmacy in the United States about Amazon Pharmacy. These findings therefore offer helpful new insights into student pharmacists’ perceptions of a changing pharmacy landscape as they prepare to enter the workforce. Each of the identified five key themes is discussed in turn below.

The first key theme was that most student pharmacists perceived positive economic outcomes for patients due to suggestions that Amazon Pharmacy can offer lower, more competitive costs. This may hold true if patients do not have insurance or have high deductibles or copayments since Amazon Pharmacy advertises that Amazon Prime members may be eligible for up to 80% off generic drugs and 40% off brand name medications [2]. If a patient does choose to use their insurance plan, it may be possible that Amazon Pharmacy mail-order services may be no different or less expensive than retail pharmacies depending on their insurance plan, medication type, and day’s supply of medication. One retrospective cross-sectional study of pharmacy claims demonstrated no difference between mail-order and community pharmacies in the overall cost per claim for 90-day quantities of maintenance medications [15]. Another study found a lower average member cost for brand drugs, off-patent brand drugs, and generic drugs from mail-order pharmacies compared to community pharmacies for patients enrolled in either of the two studied pharmacy benefit plans [16]. Similarly, a further study found lower patient costs for 90-day or greater prescriptions from mail-order pharmacies compared to retail pharmacies for patients enrolled in Medicare Part D [17]. Additional studies will need to evaluate whether patients who use Amazon Pharmacy experience lower costs with or without insurance in comparison to other mail-order pharmacies and community pharmacies.

The second key theme was that many participants predicted an improvement in medication adherence and health control, specifically for patients with chronic medication regimens. Previous research has demonstrated mail-order pharmacies can improve medication adherence for patients with diabetes on chronic medications [8]. In the current study, some participants articulated that adherence could only be as good as the patient chooses. Overall, Amazon Pharmacy is merely a tool that provides patients an opportunity to have regular access to their medications through scheduled medication deliveries with less room for gaps in therapy, thereby enabling patients to have a greater chance to improve medication adherence and subsequently health outcomes. One concern regarding the scheduled delivery of medications is possible duplications of therapy with dose or therapy changes, so it will be critical for Amazon Pharmacy to implement robust drug utilization reviews. Prospective drug use evaluations are an important aspect of pharmacist responsibilities to prevent inappropriate drug therapies and prevent adverse drug reactions [18].

Along with improved adherence, several student pharmacists perceived an improvement in health control for patients. Reasons for such improvement in health control included increased adherence and education from pharmacists via the 24/7 Amazon phone services. Such perceptions aligned with previous evaluations that demonstrated a reduction in emergency department visits and inpatient hospitalizations for patients with diabetes who use mail-order services [8] and the achievement of target low-density lipoprotein-cholesterol levels for patients new to statin therapy [7]. However, several student pharmacists commented that there could be a reduction in patient health control. Mainly, they specified that the lack of in-person intervention from a pharmacist could lead to patients incorrectly using their medications.

The third key theme, perceived humanistic outcomes for patients, was interesting because participants were split between a perceived improvement versus a perceived reduction in patients’ quality of life. Those that mentioned an improvement emphasized more convenience, more time savings, reduced transportation barriers, and decreased stress for patients who utilize Amazon Pharmacy. Those who perceived a reduction in patient quality of life mentioned a lack of personable face-to-face interactions with pharmacists with Amazon Pharmacy services. Previous work has demonstrated that patient selection of community pharmacies is influenced by positive pharmacist personalities (including traits such as friendliness, competence, and approachability) and the convenience of the pharmacy (such as wait time, hours of operation, and location) [6]. However, the patient population that Amazon Pharmacy will serve is currently unknown. Those that seek more personable interactions may not chose to use Amazon Pharmacy. Patients that select Amazon Pharmacy may indeed find many positive reasons for selecting the online/mail-order pharmacy and may find an improved quality of life from using its services. Conversely, some patients may experience a reduced quality of life if they struggle with the online interface and technology necessary to order prescriptions. The impact of Amazon Pharmacy on patient humanistic outcomes is yet to be studied.

Participants agreed that the pharmacy experience would undoubtedly be different with Amazon Pharmacy. Overall, many student pharmacists highlighted benefits of the Amazon Pharmacy experience including ease, convenience, consistency, and comfort. Perceived negative aspects of Amazon Pharmacy experience included a gap in patient–pharmacist relationships, less care for acute health issues, and less personalized service. The pharmacy experience for patients that utilize Amazon Pharmacy may include pros and cons that may be similar to the pharmacy experience for patients who receive medications from other mail-order pharmacies. Like existing mail-order pharmacies [4], Amazon Pharmacy may be more often used for chronic medications and may not be the best option for patients who need advice for acute medical problems or need a medication within a couple hours. Future studies should explore methods to improve the pharmacy experience for Amazon Pharmacy patients and evaluate factors that influence why patients use Amazon Pharmacy.

The final key theme was how Amazon Pharmacy would influence the pharmacy market. This appeared to be the most difficult topic for students to discuss. Most student pharmacists perceived that Amazon Pharmacy could expand more job opportunities for future pharmacists, while some mentioned a possible job reduction due to more automation and a desire to reduce costs and maximize efficiency. Whether Amazon Pharmacy chooses to hire future pharmacy graduates is to be determined. Overall, nearly all participants indicated that Amazon Pharmacy would have a negative impact on existing pharmacies due to competition and trust of the big company name. However, a few student pharmacists duly mentioned that where patients can receive pharmacy services may be limited based on their insurance plan and that Amazon Pharmacy may not affect other pharmacies due to services for a niche market of patients. For instance, independent pharmacies that offer compounding or medication therapy management may not be affected by Amazon Pharmacy [19,20]. Furthermore, community pharmacies may not lose too many patients if they offer medications for acute situations and services such as immunizations, smoking cessation, and diabetes management [21].

This study had some limitations. The primary limitation was that data were collected at only one college of pharmacy and therefore only represent the views of these students. Further research is needed to establish whether similar themes are identified in other, perhaps international, populations that may be of interest given the multinational nature of Amazon. Additionally, the sample most commonly had work experience within a community pharmacy setting, which could have led to more negative perceptions of Amazon Pharmacy due to the participants being partial to brick-and-mortar pharmacies. Most student pharmacists did not have previous exposure/experience with Amazon Pharmacy, so their perceptions may have been speculations based on what they know about other pharmacies, not necessarily Amazon Pharmacy. Further work with additional study populations is therefore warranted.

## 5. Conclusions

In this study, third- and fourth-year student pharmacists at one college of pharmacy offered their perceptions of how Amazon Pharmacy may affect economic, clinical, and humanistic outcomes; the pharmacy experience; and the job market. A majority perceived lower costs, improved humanistic outcomes, and improved adherence but had a mixed perception of whether health control would be improved or reduced. All student pharmacists commented that the Amazon Pharmacy experience would be different and listed several reasons why the experience may be better or worse. Participants reasoned that Amazon Pharmacy could provide either more or less job opportunities for future pharmacists and have an overall negative or unknown impact on existing pharmacies. Future studies should explore the impact of economic, clinical, and humanistic outcomes for patients that utilize Amazon Pharmacy services.

## Figures and Tables

**Table 1:** Overview of semi-structured interview core and probing questions asked to identify student pharmacist perceptions of Amazon Pharmacy.

**Patient Economic, Clinical, Humanistic Outcomes**
What is your perception of how Amazon Pharmacy will affect economic, clinical, and humanistic outcomes for patients?
Probe: Let’s look at economic outcomes first: What implications do you foresee on cost for a patient using Amazon Pharmacy versus some other pharmacy?
Probe: For clinical outcomes, what implications on patient adherence or health control do you foresee?
Probe: For humanistic outcomes, what effects on a patient’s quality of life may there be?
**Pharmacy Experience**
How do you think Amazon Pharmacy will affect the pharmacy experience?
Probe: How do you anticipate Amazon Pharmacy will impact the pharmacy experience for patients?
Probe: Can you describe what you think the patient–pharmacist relationship may look like?
**Job Market**
How do you think Amazon Pharmacy will affect the job market for pharmacists?
Probe: How do you think Amazon Pharmacy will affect opportunities for the next generation of pharmacists to find employment?
Probe: How do you think Amazon Pharmacy may affect other pharmacies, including retail chain pharmacies, mail-order pharmacies, and independent pharmacies?
**Other Perceptions**
What other perceptions of Amazon Pharmacy would you like to share with us today?

**Table 2:** Demographic characteristics of interview participants (n = 17).

Characteristics	N (%)
**Gender**	
Female	10 (59%)
Male	7 (41%)
**Previous work experience**	
Previous work experience with community pharmacy	15 (88%)
Previous work experience with hospital pharmacy	11 (65%)
Previous work experience with online pharmacy	1 (6%)
Previous work experience with medication management center	4 (24%)
Previous work experience with other type of pharmacy	2 (12%)
**Year in Pharmacy Program**	
Third-year pharmacy student	9 (53%)
Fourth-year pharmacy student	8 (47%)
**Exposure or experience with Amazon Pharmacy**	
Previous exposure or experience	5 (29%)
No previous exposure or experience	12 (71%)

## Data Availability

The data presented in this study are available on request from the corresponding author.
